# Antibody Responses to COVID-19 Vaccination in Cancer: A Systematic Review

**DOI:** 10.3389/fonc.2021.759108

**Published:** 2021-11-04

**Authors:** Deniz C. Guven, Taha K. Sahin, Saadettin Kilickap, Fatih M. Uckun

**Affiliations:** ^1^ Department of Medical Oncology, Hacettepe University Cancer Institute, Ankara, Turkey; ^2^ Department of Internal Medicine, Hacettepe University Faculty of Medicine, Ankara, Turkey; ^3^ Department of Medical Oncology, Istinye University, Istanbul, Turkey; ^4^ Department of Immunology and Inflammatory Disorders, Reven Pharmaceuticals, Westminster, CO, United States; ^5^ Immuno-Oncology Program and COVID-19 Task Force, Ares Pharmaceuticals, St. Paul, MN, United States

**Keywords:** COVID-19, vaccination, seroconversion, cancer, antibody

## Abstract

**Introduction:**

After the results of phase III vaccine studies became available, the leading oncology societies recommended two doses of COVID-19 vaccination to all patients with cancer with no specific recommendation for tumor type and active treatments. However, the data on the COVID-19 vaccine efficacy in cancer patients is limited due to exclusion of cancer patients from most vaccine clinical trials. Therefore, we systemically reviewed the available evidence evaluating the antibody responses in cancer patients.

**Methods:**

We conducted a systematic search from the Pubmed database and calculated risk differences (RD) and 95% confidence intervals (CI) to compare seroconversion rates between cancer patients and controls using the Review Manager software, version 5.3.

**Results:**

Our systematic search retrieved a total 27 studies and we included 17 studies with control arms in the analyses. Cancer patients had significantly lower seroconversion rates (37.3%) than controls (74.1%) (RD: -0.44, 95% CI: -0.52, -0.35, p<0.001) with first vaccine dose. After two doses, the seroconversion rates were 99.6% in control arm and 78.3% in cancer patients (RD: -0.19, 95% CI: -0.28, -0.10, p<0.001). The difference in seroconversion rates was more pronounced patients with hematologic malignancies (72.6%) (RD: -0.25, 95% CI: -0.27, -0.22, p<0.001) than patients with solid tumors (91.6%) (RD: -0.09, 95% CI: -0.13, -0.04, p<0.003) and patients in remission (RD: -0.10, 95% CI: -0.14, -0.06, p<0.001).

**Conclusion:**

In conclusion, COVID-19 vaccine seroconversion rates were significantly lower in patients with hematological malignancies and patients under active treatment. Further research focusing on the approaches to improve vaccine efficacy and exploration of novel treatment options is urgently needed for these patients.

## Introduction

The COVID-19 pandemic stormed the World in the last two years and caused more than four million deaths (1). Patients with cancer are among the most susceptible populations for high morbidity and mortality with COVID-19 disease (2). The increased mortality risk was especially prominent in patients with hematologic cancers, patients under active chemotherapy, and advanced age patients with additional comorbidities (3–5). The elements of the adaptive immune system, including B-cells, CD4+ T cells (especially T helper cells) and CD8+ T cells, play pivotal roles for the course, severity and health outcomes in patients with COVID-19 (6) and perturbations of the adaptive immunity have been implicated for the adverse outcomes in cancer patients with COVID-19 (7–10).

**Figure 1:**
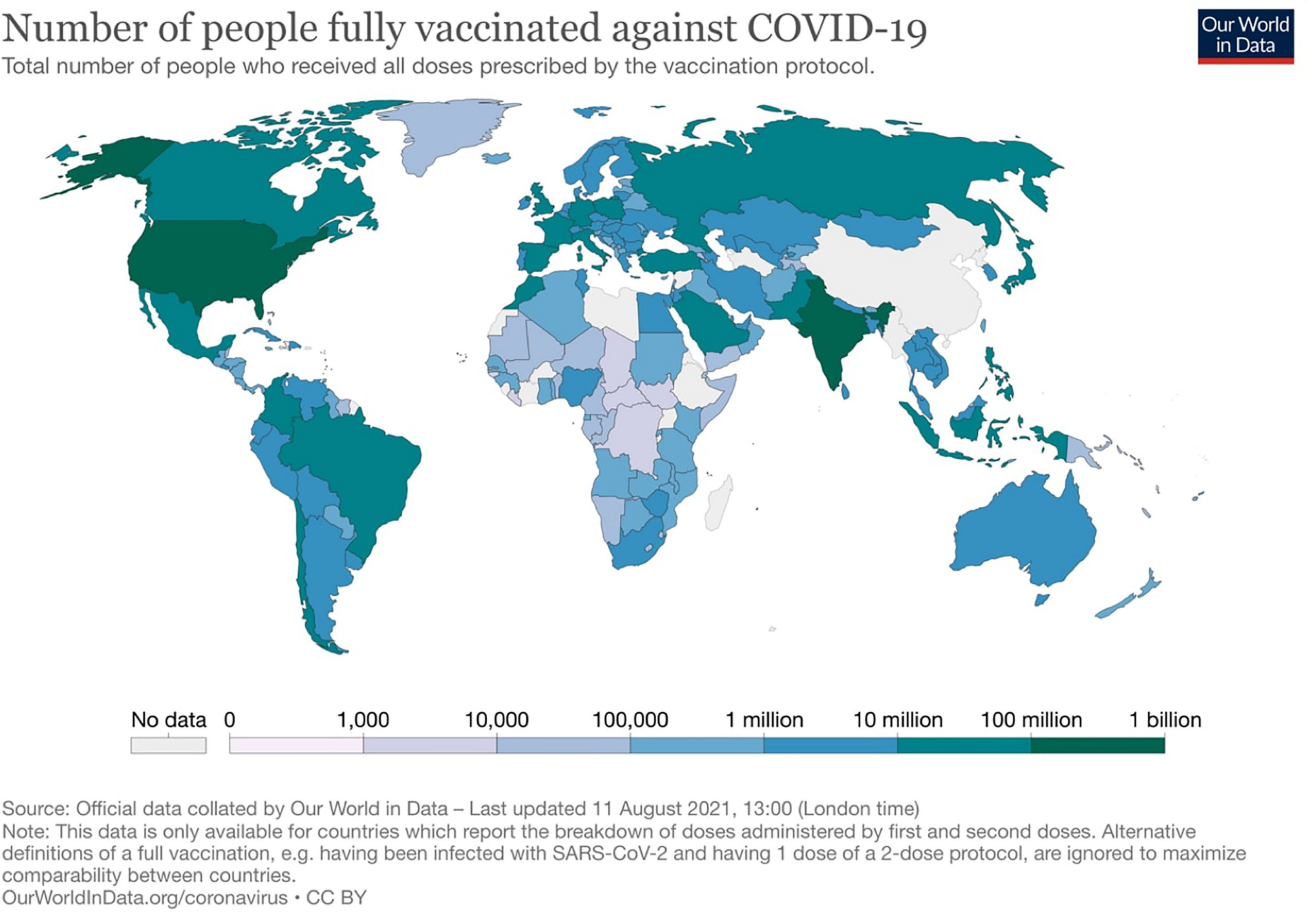
Distribution of the total number of people that have been fully vaccinated against COVID-19.

The protection of patients with cancer from COVID-19 disease while continuing optimal cancer care has been an ongoing challenge during the pandemic (11). Hopefully, the vaccination against SARS-CoV-2 showed the light at the end of the tunnel. Several vaccines, including the Pfizer/BioNTech (BNT162b2) and Moderna (mRNA-1273), exhibited safety and efficacy in large phase II and III clinical trials and received emergency approval by regulatory agencies (12–14). Almost all vaccines generated more than 90% antibody response rates and over 80% prevention rates from severe COVID-19 infection (12–14). These studies provided the foundation of a worldwide mass vaccination campaign, and to date, more than four billion doses of COVID-19 vaccines have been administered (15, 16) (**Figure 1**).

Higher case fatality rates and increased morbidity in cancer patients prompted the leading oncology groups to recommend that cancer patients should receive full COVID-19 vaccination with two doses where applicable (17, 18). However, the data on the vaccine efficacy is limited due to exclusion of cancer patients from most COVID-19 vaccine clinical trials (19). Both cancer and anti-cancer treatments challenge the proper functioning of adaptive immune machinery and could complicate the efficacy of vaccines (20). The previous experience with the influenza vaccination (21) and early reports with SARS-COV-2 vaccines (22) pointed out a decreased vaccine efficacy in patients with cancer due to both cancer and treatment-induced immunosuppression albeit with heterogeneous study populations and limited sample sizes. From these points, we systemically reviewed the available evidence of antibody responses and affecting factors for cancer patients following COVID-19 vaccination.

## Methods

### Literature Search

We conducted a systematic review from the Pubmed database per the Preferred Reporting Items for Systematic Reviews and Meta-analysis guidance (23). The MeSH search terms were “vaccine” OR “vaccination” AND “cancer” OR “malignancy” OR “lymphoma” OR “leukemia” OR “myeloma”. The search was limited to studies published between April 1st 2021 and July 26th 2021. We included the original research evaluating the seroconversion rates with SARS-COV-2 vaccines in patients with cancer and excluded reviews, opinion papers, and commentaries.

### Study Selection and Data Extraction

**Figure 2:**
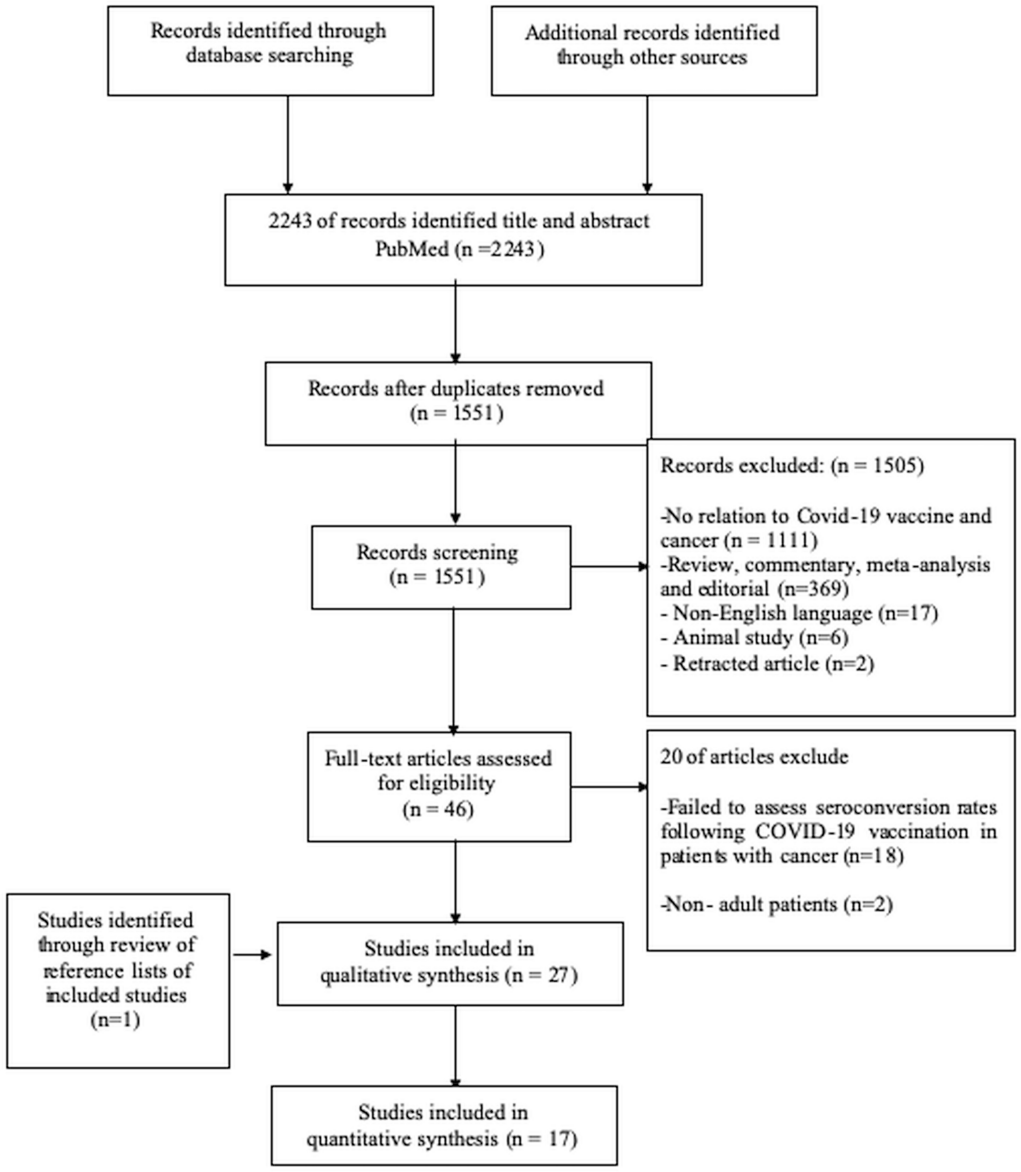
PRISMA flow diagram.

Our systematic search retrieved a total of 2243 records. After removing duplications (n=692), we screened the remaining 1551 articles. We excluded the 1505 records due to irrelevance (n=1111), reviews, commentaries, and meta-analyses (n=369), articles not in the English language (n=17), animal studies (n=6), and retracted articles (n=2). We further evaluated the remaining 46 articles and excluded 20 more records with absent details on seroconversion rates following COVID-19 vaccination in patients with cancer (n=18) and studies including pediatric patients (n=2) and included 26 records in review. One additional study was added to review from the reference lists of included studies making a total of 27 studies included in the systematic review. 17 studies with control arms were included in the quantitative synthesis (**Figure 2**).

### Meta-Analysis

We conducted separate meta-analyses to compare seroconversion rates in cancer patients and healthy controls vaccinated with one or two vaccine doses. Additionally, we conducted subgroup analyses for the malignancy type (hematologic *vs*. non-hematologic) and status of therapy (ongoing active treatment *vs*. remission off therapy). Two authors (DCG and TKS) independently reviewed and extracted the available data for the meta-analyses, and any disagreements were resolved by the senior authors (SK, FMU). We included the studies reporting seroconversion rates in the meta-analyses, while the studies with missing data for these outcomes and studies using different outcomes (i.e., antibody titers only) were excluded from the meta-analyses.

We recorded lead author names, journals, the total number of patients, seroconversion rates after one or two vaccines for each study. The risk of bias and individual study qualities was assessed independently by the DCG and TKS with Newcastle-Ottawa Scale (NOS) (**Table 1**). We performed the meta-analysis using the generic inverse-variance method with a random-effects model considering the significant heterogeneity between the studies. We selected principal summary measure as the risk differences with 95% two-sided confidence intervals to better delineate the seronegativity risk for individual patients and to prevent overestimation of seronegativity risk in cancer patients due to almost 100% seropositivity with vaccines in healthy controls. All analyses were done using the Review Manager software, version 5.3 (The Nordic Cochrane Center, The Cochrane Collaboration, Copenhagen, Denmark). The heterogeneity within each subgroup is reported using the I-square statistics. The p values below 0.05 were considered statistically significant.

**Table 1:** Newcastle-ottowa scores of included studies.

Author, year	Selection	Comparability	Exposure/Outcome	Reference
Herishanu Y, Blood	***	**	***	(24)
Massarweh A, JAMA Oncol	****	**	***	(25)
Palich R, Ann Onc	***	**	***	(22)
Oekelen OV, Cancer Cell	**	**	**	(26)
Barrière J, Ann Onc	**	**	**	(27)
Monin L, Lancet Oncol	****	**	***	(28)
Pimpinell F, J Hematol Oncol	***	**	**	(29)
Avivi I, Br J Haematol	****	**	***	(30)
Tzarfati KH, Am J Hematol	****	**	***	(31)
Shroff RT, MedRxiv	****	**	***	(32)
Addeo A, Cancer Cell	***	*	**	(33)
Goshen-Lago T, Jama Oncol	****	**	***	(34)
Palich R, Ann Onc 2	***	**	**	(22)
Gavriatopoulou M, Clin Exp Med	****	**	***	(35)
Terpos E, Journal of Hematology & Oncology	***	**	**	(36)
Chowdhury O, Br J Haematol	**	**	***	(37)
Terpos E, Blood	***	**	***	(38)

The stars refer to the scores.

## Results

### Study Characteristics

A total of 27 studies evaluated the seroconversion rates after at least one dose of a COVID-19 vaccine. The sample sizes were very variable (minimum 16- maximum 423). Most studies (18/27) included a control group which involved mostly health care workers. Seven studies included only patients with solid tumors (22, 25, 27, 32, 34, 36, 39), four studies included both solid tumor patients and patients with hematologic malignancies (28, 33, 40, 41) and 16 studies included only patients with hematologic malignancies (24, 26, 29–31, 35, 37, 38, 42–49). Nine studies included both patients in remission and patients under active treatment. Twelve studies measured baseline anti-SARS-CoV-2 antibodies and excluded patients with positive baseline antibody titers (27–30, 33, 37–39, 41, 44, 48, 49).Eight studies used only previous COVID-19 history as the exclusion criteria (24, 25, 31, 34–36, 43, 46)and 4 studies included patients with previous COVID-19 history (22, 26, 40, 42). The antibody measurement methods were very heterogenous between the studies and immunoglobulin G (IgG) antibodies against the SARS-CoV-2 spike protein were the most commonly used antibody assay (**Table 2**).

**Table 2:** Summary of studies evaluating antibody responses to SARS-CoV-2 vaccination.

Lead Author, Journal	Patient Cohort	Healthy Control	Number of Participants	Baseline Antibody Measurement	Antibody Assay	Platform	Vaccine	Seroconversion Rate After 1st Dose	Seroconversion Rate After 2nd Dose	Seroconversion Rate of Control Group	Additional Findings	Reference
Massarweh A, JAMA Oncol	Solid Tumors	Y	102 Patient/78 Control	N/A	SARS-CoV-2 IgG II	Abbott (architect i2000sr platform)	BNT162b2	N/A	90%	100%	Lower antibody titers in patients treated with chemotherapy plus immunotherapy (p=0.001)	(25)
				(No History of COVID-19)	Quant assay							
Herishanu Y, Blood	CLL	Y	167 Patients (52 Patient and 52 Control for Matched Cohort)	N/A	Anti-SARS-CoV-2 S	Elecsys (Analyzer: Cobas E 601)	BNT162b2	N/A	39.5%	100%	Lower seroconversion in patients under treatment (16%) *vs*. patients with clinical remission (79.2%) and treatment-naïve patients (55.2%)/No seroconversion in patients exposed to anti-CD20 treatment in last 12 months (0/22)	(24)
				(No History of COVID-19)								
Palich R, Ann Onc	Solid Tumors	Y	110 Patients/25 Controls	N/A	Serum	Abbott	BNT162b2	55%	N/A	100% after 1^st^ dose	Lower seropositivity in >65 years (OR: 3.58, 95% CI: 1.40-9.15, p=0.008), and treatment with chemotherapy (OR: 4.34, 95% CI: 1.67-11.30, p=0.003)	(22)
				(Prior COVID-19 infection in 15 patients as evidenced by positive anti-N IgG)	anti-N IgG and anti-S IgG							
Palich R, Ann Onc	Solid Tumors	Y	223 Patient/49 Control	Negative anti-SARS-CoV-2 anti-nucleoprotein IgG	SARS-CoV-2 immunoglobulin (Ig) G/SARS-CoV-2	Abbott Alinity/Roche Elecsys	BNT162b2	N/A	94%	100%	8/13 of the seronegative patients had metastatic disease and 10/13 seronegative patients were treated with chemotherapy	(39)
					total Ig electrochemiluminescent immunoassay							
Barrière J, Ann Onc	Solid Tumors	Y	122 Patients/31 Controls	Negative SARS-CoV-2 S	Anti-SARS-CoV-2 S	Elecsys	BNT162b2	47.5%	95.2%	100%	Patients under active	(27)
											CT lower seroconversion rates after first-dose compared to patients without CT, and patients under targeted therapy alone (42.9% *vs*. 76.5%, p=0.016)	
Goshen-Lago T, Jama Oncol	Solid Tumors	Y	232 Patients/261 Controls	N/A	SARS-CoV-2 anti-spike (S) S1/S2 IgG assay	Liaison^®^ analyzer	BNT162b2	29%	86%	84% after 1^st^ dose	Low rates of seropositivity in patients undergoing chemotherapy	(34)
				(No History of COVID-19)							(OR: 0.41, 95%CI: 0.17-0.98)/Low rate of systemic adverse events	
Shroff RT, medRxiv	Solid Tumors	Y	52 Patients/50 Controls	Neutralizing antibodies (WA1 isolate)	Neutralizing antibodies (WA1 isolate)	ImmunoSpot Versa	BNT162b2	67%	80%	98% after 1^st^ dose/ 100%	Lower overall antibody and T cell responses in cancer cohort compared with control cohorts	(32)
										after 2^nd^ dose		
Terpos E, J Hematol Oncol	Cancer patients receiving checkpoint inhibitors	Y	59 Patients/283 Controls	N/A	Neutralizing antibodies (SARS-CoV-2 NAbs Detection Kit)	cPass™	BNT162b2 and mRNA-1273	25%	N/A	65.7	None of the patients enrolled had neutropenia or lymphopenia at first vaccination dose	(36)
				(No History of COVID-19)								
Addeo A, Cancer Cell	Solid/Hematologic Malignancies	N	131	Negative anti-SARS-CoV-2 nucleocapsid	Anti-SARS-CoV-2 S	Elecsys	BNT162b2 and mRNA-1273	83% in solid tumors/77% in hematological tumors	94%	N/A	Lower seroconversion in hematological malignancy (77% *vs*. 98%)/Lower seroconversion in patients treated with chemotherapy or immunotherapy (98% *vs*. 93% and 93%)/No seroconversion under anti-CD20 treatment (0/4)	(33)
				protein IgG								
Monin L, Lancet Oncol	Solid/Hematologic Malignancies	Y	151 Patients/54 Controls	Negative SARS-CoV-2 S/Negative rRT-PCR	SARS-CoV-2 S-specific IgG	Local (ELISA)	BNT162b2	38% solid tumor/18% hematologic cancer	95% solid tumor/60% hematologic cancer	94% after 1^st^ dose/100% after 2^nd^ dose	No adverse events in more than 50% of the patients with vaccination/T-cell responses in 82%, 71% and 50% of the controls, solid tumor cohort and hematologic tumor cohort with first vaccine dose	(28)
Thakkar A, Cancer Cell	Solid/Hematologic Malignancies	N	200	N/A	SARS-CoV-2 IgG II	AdviseDx CIMA (on Abbott i2000SR instrument)	BNT162b2, mRNA-1273 and Ad26	N/A	94%	N/A	Lower seroconversion rates in hematologic malignancies (85%), patients treated with anti-CD20 therapies (70%) and stem cell transplantation (73%) compared to patients with solid tumors (98%)/Higher seroconversion rates in patients treated with immunotherapy (97%) or hormonal therapies (100%)	(40)
				(*Prior COVID-19 infection in 18 patients)								
Fong D, Eur J Cancer	Solid/Hematologic Malignancies	N	154	Negative Anti-SARS-CoV-2 nucleocapsid and spike protein IgG	SARS-CoV-2	Abbott	BNT162b2	61%	85.7%	N/A	Higher antibody titres in *CoV-positive* patients after the first vaccine dose (p<0.001)	(41)
					nucleocapsid and spike protein IgG chemiluminescent immunoassay							
Oekelen OV, Cancer Cell	MM	Y	320 Patients/67 Controls	N/A (Prior COVID-19 infection in 60 patients)	SARS-CoV-2 IgG test	COVID-SeroKlir Kantaro	BNT162b2 and mRNA-1273	N/A	84.2%	100%	Lower seroconversion rates in patients treated with anti-CD38 (HR: 4.258, p=0.005) or BCMA-targeted treatment (HR: 10.269, p<0.001)/Better seroconversion rates in patients with CR (HR: 0.389, p=0.037)	(26)
Bird S, Lancet Haematol	MM	N	93	N/A	Anti-SARS-CoV-2 IgG and AntiSARS-CoV-2 total antibody against S1 spike protein	Ortho Clinical Diagnostics	BNT162b2 and AZD1222	56% (70% total antibody response)	N/A	N/A	Higher seroconversion in responding patients (p=0.0046)/Lower seroconversion rates in patients under treatment (48% *vs*. 74%, p=0.037)/Similar seroconversion rates with Pfizer and AstraZeneca vaccines	(42)
Pimpinell F, J Hematol Oncol	MM/MPN	Y	92 Patients/36 Controls	SARS-CoV-2 S1/S2 IgG test	SARS-CoV-2 S1/S2 IgG test	Liaison^®^	BNT162b2	21.4% in myeloma/52% in MPN	78.6% in myeloma/88% in MPN	52.8% after 1^st^ dose/100% after 2^nd^ dose	Lower seroconversion rates in daratumumab-treated patients (50% *vs*. 92.9%, p=0.003)/Low rate of systemic adverse events	(29)
						analyzer						
Terpos E, Blood	MM	Y	48 Patients/104 Controls	Neutralizing	Neutralizing	cPass™	BNT162b2	25%	N/A	54.8% after 1^st^ dose	All patients with clinically relevant viral inhibition (%4/4) after first dose was in remission without treatment	(38)
				Antibodies Against SARS-CoV-2	Antibodies Against SARS-CoV-2							
Lim SH, Lancet Haematol	Lymphoma	Y	119 Patients/150 Controls	Negative anti-SARS-CoV-2 nucleocapsid	Qualified electrochemiluminescent Anti-SARS-CoV-2 S assay	Meso Scale Discovery	BNT162b2 and AZD1222	N/A	N/A	100%	Lower seroconversion rates in patients who received systemic anti-lymphoma therapy after the first vaccine dose (p= 0.0002), after the second vaccine dose p<0.001 for BNT162b2 vaccine)	(44)
				protein IgG								
Avivi I, Br J Haematol	MM	Y	171 Patients/64 Controls	Negative anti-SARS-CoV-2 nucleocapsid	Anti-SARS-CoV-2 S	Elecsys	BNT162b2	N/A	78%	98%	Lower seroconversion rates in daratumumab-treated patients (69% *vs*.81%, p=0.08)	(30)
				protein IgG								
Chowdhury O, Br J Haematol	CML	Y	59 Patients/232 Controls	Negative anti-SARS-CoV-2 nucleocapsid	SARS-CoV-2 IgG II Quant Assay	Abbott	BNT162b2 or AZD1222	58%	N/A	97% after 1^st^ dose	The highest seroconversion rates in patients with CML (75%)	(37)
				protein IgG								
Roeker LE, Leukemia	CLL	N	44	N/A	SARS-CoV-2 S1/S2 IgG assay	Liaison^®^	BNT162b2 and mRNA-1273	N/A	52%	N/A	Lower seropositivity in >70 years (OR: 12, 95% CI: 2.9-50.5, p=0.001), and prior-CLL directed therapy (OR: 56.7, 95% CI: 6.2-518, p<0.001)	(45)
Agha M, medRxiv	Hematologic Malignancies	N	67	N/A	Semi-quantitative SARS-CoV-2 IgG against the Spike protein receptor-binding domain	Beckman Coulter SARS-CoV-2 platform	BNT162b2 and mRNA-1273	N/A	53.7%	N/A	Lower seroconversion in CLL patients compared to patients with other hematological malignancies (23.1% *vs* 61.1%, p = 0.01)	(46)
				(No History of COVID-19)								
Diefenbach C, medRxiv	CLL, HL and NHL	Y	53 Patients/5 Controls	N/A	Multiplex bead-binding IgG spike and receptor binding domain assay for SARS-CoV2	Yeti ZE5 Cell	BNT162b2 and mRNA-1273	47.1%	N/A	100%	Lower seroconversion rates in patients treated with anti-CD20 (p<0.001) and BTK inhibitors (p=0.003)/No effect of additional boost on antibody titers in most patients (94%)	(47)
						Analyzer						
Gavriatopoulou M, Clin Exp Med.	WM, CLL and NHL	Y	58 Patients/213 Controls	N/A	Neutralizing antibodies	cPass™	BNT162b2 and AZD1222	14%	N/A	%54	Lower response rates (< 30%) in patients under active treatment	(35)
				(No History of COVID-19)								
Tzarfati KH, Am J Hematol	Hematologic Malignancies	Y	315 Patients/108 Controls	N/A	SARS-CoV-2 S1/S2 IgG test	Liaison^®^	BNT162b2	N/A	75%	99%	Older age (p< 0.001), higher lactate dehydrogenase (p=0.02), and number of treatment lines (p< 0.001) was correlated with lower seropositivity	(31)
				(No History of COVID-19)								
											Absolute lymphocyte count (p 0.001), total globulin level (p=0.002), and time from last treatment to vaccination(p<0.001) correlated with higher seropositivity likelihood and antibody titers	
Harrington P, Leukemia	CML	N	16	Negative anti-SARS-CoV-2 anti-nucleoprotein IgG	SARS-CoV-2 Anti-S IgG ELISA	Local	BNT162b2	81.25%	N/A	N/A	Higher post-vaccine anti-S IgG EC50 and neutralising antibody ID50 titres in myelofibrosis patients (n = 9) compared to patients with other MPN subtypes (p = 0.012)	(48)
Harrington P, Br J Haematol	CML	N	16	Negative Anti-SARS-CoV-2 nucleocapsid and spike protein IgG	SARS-CoV-2 Anti-S IgG ELISA	Local	BNT162b2	87.5%	N/A	N/A	No statistical difference seen between diffrent TKIs in neutralising antibody titres (p=0.68)	(49)
Re D, Leuk Lymphoma	Hematologic Malignancies	N	102	N/A	Commercially avaible kit detecting SARS-CoV-2 anti-spike (S)	N/A	BNT162b2 and mRNA-1273	N/A	62.7%	N/A	Lower seroconversion rates after the first vaccine dose in patients who received anti-CD20 treatment beyond the last 12 months (p< 0.0001)	(43)
				(No History of COVID-19)								

ALC, Absolute lymphocyte count; BCMA, B cell maturation antigen; BTK, Bruton tyrosine kinase, CML, Chronic myeloid leukemia; CLL, Chronic lymphocytic leukemia; COVID-19, Coronavirus disease 2019; HL, Hodgkin lymphoma; CT, Chemotherapy; MM, Multiple myeloma; MPN, Myeloproliferative neoplasms; N/A, Not available; NHL, Non-Hodgkin lymphoma; RBD, receptor binding domain; SARS-CoV-2, Severe Acute Respiratory Syndrome Coronavirus-2; WM, Waldenstrom macroglobulinemia.

### Seroconversion Rates After First Vaccination

**Figure 3:**
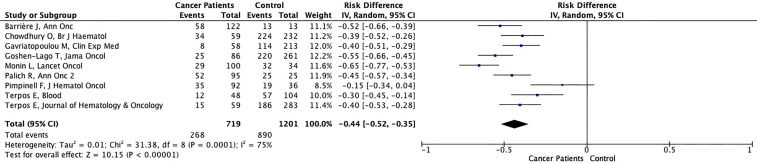
Forest plot illustrating the risk differences of seroconversion rates between cancer patients and healthy controls with first dose of vaccination.

Seroconversion rates after the first dose of vaccination and second dose of vaccination were reported in 17 studies each. Seven studies reported seroconversion rates after both the first and second vaccine doses (27–29, 32–34, 41). Low seroconversion rates after the first vaccine dose were consistent across all studies, and the reported seroconversion rates were only around 20-30% for patients with lymphoid malignancies (**Table 2**). After the first vaccine dose, the seroconversion rates were quite variable in patients with solid tumors and ranged between 29% to 83%. After the first dose of vaccination, the seroconversion rates of control groups were over 90% in all but 4 studies. The 4 studies with lower seroconversion rates in the control group after the first vaccine dose used mostly octogenerians as control group (29, 36, 38). In the pooled data from 9 studies with control arms, the possibility of seroconversion was significantly lower in cancer patients (268/719, 37.3%) than healthy controls (890/1201, 74.1%) after first dose of vaccination (RD: -0.44%, 95% CI: -0.52%, -0.35%, p<0.001) (**Figure 3**). Significant variability existed among the studies (I^2 =^ 75%) (**Figure 3**). Six studies included only patients vaccinated with the BNT162b2 vaccine, while two studies included both the BNT162b2 or AZD1222 vaccines (35, 37) and one study included patients vaccinated with either of the mRNA vaccines (BNT162b2 and mRNA-1273) and AZD1222 vaccine (36). Further analyses with the exclusion of studies including vaccines other mRNA vaccines (seven studies), demonstrated a consistent risk of seronegativity in cancer patients compared to controls (RD: -0.45%, 95% CI: -0.58%, -0.33%, p<0.001) (Supplement). Similarly, in the pooled analyses of three studies (35–37) including both mRNA and AZD1222 vaccines, the seroconversion rates were significantly lower in cancer patients (RD: -0.40%, 95% CI: -0.47%, -0.33%, p<0.001) (Supplement). The separate data on the seroconversion rates with mRNA and AZD1222 vaccines was only available in one study. The seroconversion rates were similar for AZD1222 (59.5%) and BNT162b2 (54.5%) vaccines in this study after the first dose of vaccination (37).

### Seroconversion Rates After Second Vaccination

In contrast to low seroconversion rates after the first dose of vaccination, patients with solid tumors who received their complete vaccination had seroconversion rates greater than 90%. Likewise, patients with hematologic malignancies had over 75% seroconversion rates in all but one study (24). Additionally, anti-CD20 treatments in lymphoma patients and anti-CD38 treatments in multiple myeloma patients were associated with low seroconversion rates in 5 (24, 33, 40, 43, 47) and 3 studies (26, 29, 30), respectively. By comparison, the seroconversion rates almost 100% in the control arms with complete vaccination in all reported studies (538/540, 99.6%).

**Figure 4:**
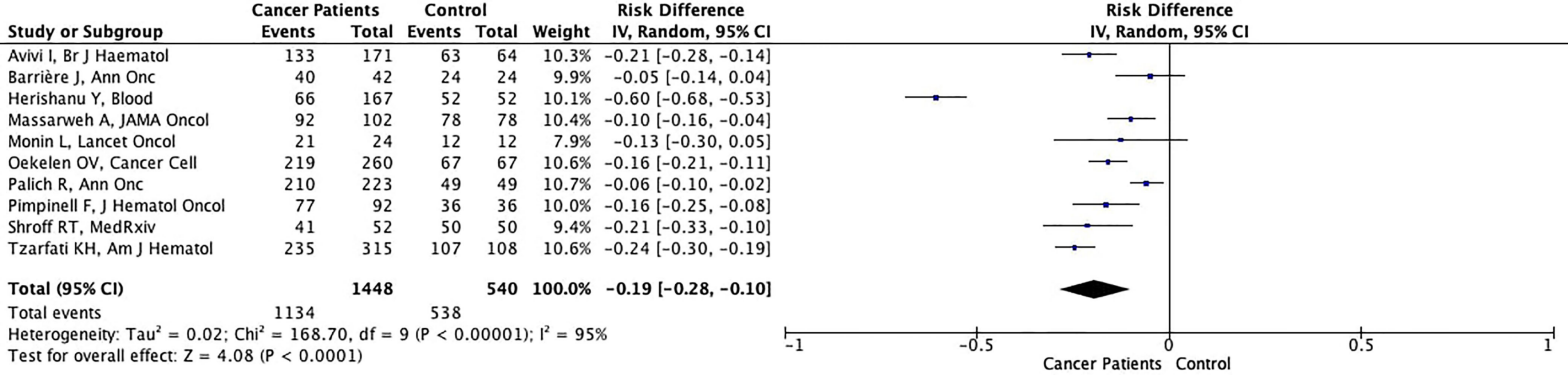
Forest plot illustrating the risk differences of seroconversion rates between cancer patients and healthy controls after second dose of vaccination.

**Figure 5ABC:**
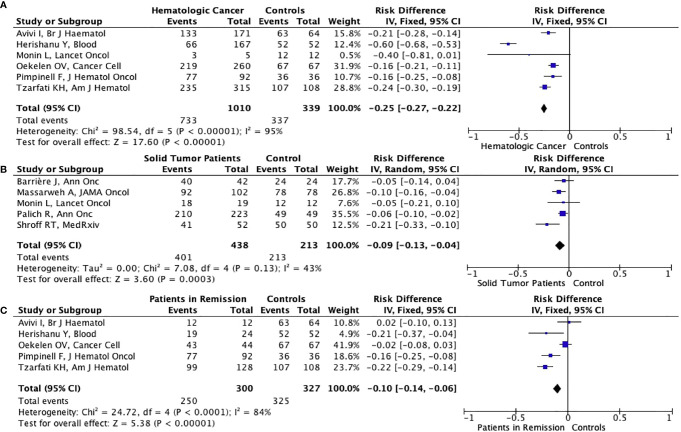
Forest plot illustrating the risk differences of seroconversion rates between patients with hematologic malignancies **(A)**, solid tumors **(B)** or patients in remission **(C)** and healthy controls after second dose of vaccination.

The possibility of achieving seroconversion was 19% lower in cancer patients (78.3%) compared to controls (99.6%) (RD: -0.19%, 95% CI: -0.28%, -0.10%, p<0.001), in the pooled data of ten studies with available seroconversion rates after complete vaccination (**Figure 4**). The antibody titers were lower in cancer patients than controls in most studies including control arms (**Table 2**). In contrast, Goshen-Lago (34) and Addeo et al. (33) reported similar neutralizing anti-SARS-CoV-2 antibody titers in patients with active cancer and patients whose cancer is in remission, respectively. The difference in seroconversion rates was more pronounced for patients with hematologic malignancies (733/1010, 72.6%) (RD: -0.25%, 95% CI: -0.27%, -0.22%, p<0.001) (**Figure 5A**) than patients with solid tumors (401/438, 91.6%)

(RD: -0.09%, 95% CI: -0.13%, -0.04%, p<0.003) (**Figure 5B**). Additionally, six studies (five involving only patients with hematologic malignancies) reported specific outcomes for patients in remission. In the pooled analysis of 5 studies with control arms, the cancer patients in remission had significantly lower seroconversion rates than healthy controls albeit with a smaller risk difference (RD: -0.10%, 95% CI: -0.14%, -0.06%, p<0.001) (**Figure 5C**). Significant heterogeneity was present in all analyses other than analyses in the solid tumor subgroup (I^2 =^ 43%) (**Figures 4**, **5A–C**). Nine of the ten studies included patients vaccinated with the BNT162b2 vaccine, while the study by Oekelen et al. (26)included patients vaccinated with the BNT162b2 or mRNA-1273 vaccine. Due to use of mRNA vaccines in all of the studies, no stratification could be done according to vaccine type.

## Discussion

To the best of our knowledge, this was the first meta-analysis on the antibody responses to COVID-19 vaccination in cancer patients. In the pooled analyses of the studies, cancer patients had significantly lower seroconversion rates with one or two doses of vaccination. The seroconversion rates were especially lower in patients with hematologic malignancies, while patients with solid tumors and patients in remission had slightly reduced antibody responses to vaccination.

Vaccination against SARS-CoV-2 is vital for patients with cancer. However, T-cell immunity is severely impaired in most cancer patients which could reduce the immune responses to vaccines (50). The first clue of this problem was evident in the study by Solodky et al. reporting significantly lower seroconversion rates in cancer patients after SARS-CoV-2 infection (30% in cancer patients *vs*. 71% in health care workers, p=0.04) (51). However, the sample size was very small (n=24) (51). Marra et al. challenged this finding in a larger cohort (n=166) and reported similar seropositivity between cancer patients (80.5%) and health care workers (87.9%) after COVID-19 infection (p=0.39) (52). Later, Takkar and colleagues demonstrated a high seroconversion rate (92%) in the 261 patients with cancer after COVID-19 disease, although significantly lower seroconversion rates in patients with hematological malignancies (82%) and patients who received anti-CD20 treatment (59%) were concerning (53).

Cancer patients were among the prioritized groups of persons for COVID-19 vaccination (18). However, the data on the efficacy of vaccines were limited in cancer patients due to the exclusion of these patients from clinical trials. This issue made studies from real-world evidence settings imperative and led to a continuous effort in that direction. The earliest studies evaluated the seroconversion rates after the single vaccine dose and focused on the hematological cancers as a susceptible group for low seroconversion rates. Single-dose mass vaccination, generally with a prolonged delay for the second dose, was proposed to provide a broader vaccine coverage due to the limited vaccine supply (15). Initial reports reported over 70% protection from symptomatic COVID-19 disease with a single dose vaccine in the general population (54, 55) and this observation created the foundation of extended interval vaccination in England to vaccinate a larger part of the population with at least one vaccine dose. However, this strategy seems risky and not sustainable for cancer patients considering their already significantly lower seroconversion rates with first vaccine dose (37.3% in cancer patients *vs*. 74.1% in controls, **Figure 3**). Antibody titers were also significantly lower with a single vaccine dose in seroconverted patients (22, 29, 33), necessitating the application of a second dose vaccine in a time interval recommended in clinical trials.

Although the seroconversion rate for cancer patients was increased after the second dose of the vaccine, the rate was still significantly lower than the controls. Patients with solid tumors had over 90% seroconversion rates, while the patients with hematologic malignancies had significantly lower seroconversion rates (**Table 2**). The lower seroconversion rates were especially prominent in patients with lymphoid malignancies (**Table 2**). Additionally, patients treated anti-B cell antibodies targeting CD20 or CD38 antigens as part of their standard of care had significantly lower antibody responses after COVID-19 vaccination (26, 33). The negative effect of anti-CD20 therapy appeared to be long-lasting, as evidenced by lack of seroconversion in 22 chronic lymphocytic leukemia patients who were treated with anti-CD20 antibodies within the last 12 months (24). We think that these patients should be prioritized for novel approaches for COVID-19 like anti-SARS-COV-2 antibody studies (56) and third dose vaccinations (57).

An early report in 30 solid organ transplant recipients with negative or low antibody levels after two vaccination doses, an additional vaccine boost increased antibody titers in all patients with low antibody response (6/6) and created seroconversion in the 6 of 24 seronegative patients. 80% of the study population were received boosts with a different vaccine in the study (58). An anecdotal report by Hill et al. also supported the possible benefit of an additional boost with a heterologous vaccine providing seroconversion in a seronegative lymphoma patient (59). Recently, a phase II study with CoronaVac vaccine in healthy adults reported significantly increased antibody titers with a third dose boost which applied 6-8 months after the second dose in patients became seronegative (60). If further research supports these observations, the seronegative patients with cancer could benefit from a three-dose vaccination schedule similar to strategies with influenza and hepatitis B vaccinations (61, 62). In contrast to those observations, a small study on 18 seronegative CLL, NHL and myeloma patients with two doses of BNT162b2 vaccination demonstrated no seronconversion with a third dose (57). A large phase I study is currently evaluating the benefit of a three-dose vaccination in 1000 patients with cancer (NCT04936997) and hopefully could aid to determine the best strategy in seronegative cancer patients. While the oncology community is eagerly awaiting the results of three-dose vaccination studies, the Centers for Disease Control and Prevention (CDC) recommended a third-dose Pfizer/BioNTech (BNT162b2) and Moderna (mRNA-1273) vaccine booster in solid organ transplantation patients or who have a similar level of immunosuppression based on the two studies in transplantation patients demonstrating more than a 30% increase in seroconversion rates with a third-dose boost (63, 64). This immunosuppression definition includes cancer patients under active treatment or patients who had stem cell transplantation in the last two years and these patients should have a third-dose booster until further data is available (65). Several other countries including Italy and Turkey also recommended a third-dose booster in older patients and patients with comorbidities creating immunosuppression. However, data on the booster dose efficacy in immunosuppressed patients is not available for other vaccines including inactive whole virus vaccines and viral vector vaccines.

There are still significant knowledge gaps and unanswered questions. The seroconversion is often used as a surrogate laboratory marker of adaptive immunity against vaccination in immunocompromised cohorts (29, 66). However, the available studies have yet to report any meaningful correlation between measured anti-SARS-Cov-2 antibody titers and T-cell immunity against COVID-19 in cancer patients following vaccination. T-cells are the main actors of the fight against COVID-19 and the creation of the long-lasting immune memory against COVID-19 (67) and could be a better reflector of the immunity against COVID-19. A recent report with the BBIBP-CorV vaccine reported no significant correlation between the serum IgG antibody titers and interferon-gamma concentrations in the peripheral blood mononuclear cells (p=0.11) (68) suggesting antibody measurements could be imprecise to detect SARS-CoV-2 specific adaptive immunity. The imprecision of seroconversion as a COVID-19 vaccine efficacy was further supported by the work of Tzarfati and colleagues. The authors reported no COVID-19 cases in 315 patients with hematologic malignancies vaccinated with two doses of BNT162b2 vaccine, albeit with a relatively low seroconversion rate (75%) in the cohort pointing out the limitation of seroconversion rate as the sole denominator of immunity (31). The evaluation of T-cell immunity with vaccination is being addressed in the SOAP-02 study (28) and could be especially important in patients with low or negative antibody responses to vaccination (69). In addition to the one-dimensional nature of antibody measurements as a denominator of vaccine efficacy, the SARS-CoV-2 antibody assays have previously been reported to suffer from moderate concordance and variable sensitivities. These inherent limitations have emerged as an important challenge in accurately diagnosing the SARS-CoV-2 infection during the early phases of pandemic (70–72). Several new strategies including the combined use of ELISA and virus neutralization tests have been proposed to improve the diagnosis of COVID-19 by increasing the sensitivity of detection for SARS-CoV-2 (73). The diagnostic challenges have resolved with with the rapid development of assays with significantly improved sensitivity and specificity (74). While several studies have previously evaluated the vaccine seroconversion rates based on antibody assays with lower sensitivities (29, 31) or antibody assays without an FDA authorization developed for research purposes (32), a consistent trend across studies and the use of Elecsys and Abbott spike IgG assays (>95% sensitivity and specificity for both) (**Table 2**), have decreased the possibility of confounding problems due to antibody assay performance during the evaluation of seroconversion rates with COVID-19 vaccination.

Furthermore, B-cell immunity against COVID-19 is hampered by emergence of variants of concern, such as B.1.1.7 (alpha strain from UK), B1.351 (beta strain from South Africa), B.1.617.2 (delta strain from India) and P.1 (gamma strain from Brazil) characterized by mutant spike proteins, that may not be effectively neutralized by low titers of anti-SARS-CoV-2 spike protein antibodies induced by available COVID-19 vaccine platforms using the ancestral strain of SARS-CoV-2. A recent study from the United Kingdom reported modestly decreased vaccine efficacy against delta variant, especially with a single-dose vaccination (75). The authors including 316 participants from the clinically extremely vulnerable group, including cancer patients. However, a separate dataset was not available for cancer patients (75). The results of COVIVAC-ID (NCT04844489) and EREVA (NCT04952766) studies are eagerly anticipated to delineate the vaccine efficacy against variants of concern in cancer patients.

Another knowledge gap pertains to the effects of different anti-cancer treatments on antibody responses to COVID-19 vaccination, especially immunotherapy and chemotherapy. Previous studies have demonstrated that cancer patients treated with immune checkpoint inhibitors (ICIs) or precision medicines (e.g. tyrosine kinase inhibitors) who subsequently develop COVID-19, can enjoy survival outcomes that are similar to the survival outcomes of the general population with COVID-19 (3, 76). It’s hypothesized that cancer patients treated with ICI or precision medicines have significant T-cell immunity against viral infections (76, 77). The retained T-cell immunity could also orchestrate efficient responses to vaccination (78), similar to the robust antibody responses to COVID-19 vaccination in myeloma patients with treated with immunomodulatory agents (79). However, in part due to a focus on hematological cancers and the lack of separate studies for seroconversion rates, the published information regarding the seroconversion rates for cancer patients treated with ICI or precision medicines is very limited and the data is unequivocal. Addeo et al. reported similar seroconversion rates (93% in both) in ICI- *versus* chemotherapy treated patients. But the median antibody titers (1.116 *vs*. 611 U/mL) and seroconversion rates after the first vaccine dose (85 *vs*. 69%) were significantly higher in ICI-treated patients compared to patients treated with cytotoxic chemotherapy. Additionally, 21 of the 22 patients treated with anti-HER2, anti-VEGF, RANKL inhibitors or kinase inhibitors had reportedly seroconversion after two vaccine doses (33). Similarly, Thakkar et al. reported 97% and 100% seroconversion rates in patients treated with ICI and hormonal therapy, respectively (40). Singer et al. reported higher seroconversion rates in ICI-treated patients compared to patients treated with chemotherapy, targeted therapy or radiotherapy. Additionally, the seroconversion rates were higher with the ICI-chemotherapy combinations than patients treated with chemotherapy only, supporting a possible benefit of ICIs in immune response against vaccination (80). In contrast, Massarweh et al. pointed out a possible adverse synergistic effects of combined ICI + chemotherapy or biotherapy on COVID-19 antibody responses and reported significantly lower median antibody titers in patients treated with combinations of ICI and chemotherapy or biotherapy than patients treated with chemotherapy alone (25). Similarly, Terpos et al. reported significantly lower seroconversion rates after first vaccination dose in 59 ICI-treated patients compared to healthy controls (25 *vs*. 65.7%, p<0001) (36). These unequivocal findings emphasize the need for additional studies focusing on these patients. Similarly, whether antibody responses were impaired in patients treated with radiation therapy remains to be deciphered.

An important point is the paucity of data on the optimal vaccination schedule in cancer patients with COVID-19 history. While these patients are vaccinated with schedules similar to general population as seen in the reported studies (**Table 2**), Reynolds et al. reported robust T and B-cell responses against B.1.1.7 variant with a single boost of mRNA vaccine in 25 patients with COVID-19 history (81). Similarly, Fong et al. reported significantly higher seroconversion rates in cancer patients with COVID-19 history (n=89) compared to patients without COVID-19 history (n=154) (91% *vs*. 61%) (41). Whether a single dose vaccination strategy could be suitable in patients with COVID-19 history should investigated in larger cohorts. Further, it is unknown whether available vaccines differ in protecting cancer patients from symptomatic or severe COVID-19. The available studies were mostly conducted with mRNA vaccines, and Addeo et al. reported similar seroconversion rates with two different mRNA vaccines (33), while Lim et al. reported similar antibody responses to BNT162b2 and ChAdOx1 vaccines in lymphoma patients (44). While the mRNA and adenovirus vectors attach to different toll-like receptors (TLR7 and TLR9, respectively), after the TLR attachment both vaccines cause the type I interferon secretion and the activation of the CD4-positive T-cells. This mechanism of action suggests both vaccines could have a similar efficacy from a biologic standpoint (82). However, several parts of the World, including China, Russia, and India, use different vaccines. Reports from different parts of the World will be critically important and could direct the optimal vaccination planning for cancer patients in the future. Studies with the different vaccines from these countries, and separate reporting for seroconversion rates with different vaccines is vital for the future studies and vaccination planning in cancer patients.

In conclusion, patients with cancer had significant seroconversion rates with a two-dose vaccination schedule, while seroconversion rates were significantly lower in patients with hematological malignancies and patients under active treatment. Given the life-saving nature of anti-cancer treatments, further research focusing on these patients is urgently needed.

## Data Availability Statement

The original contributions presented in the study are included in the article/**Supplementary Material**. Further inquiries can be directed to the corresponding author.

## Author Contributions

DG and FMU have planned the work. DG, TS, SK and FMU participated in data collection. All authors have made significant and substantive contributions to the reporting of the work, drafting of the manuscript, review and revisions of the final draft. All co-authors qualify the criteria for authorship according to Vancouver protocol.

## Conflict of Interest

FMU was employed by Ares Pharmaceuticals, LLC and was a consultant for Aptevo Therapeutics and for Reven Pharmaceuticals.

The remaining authors declare that the research was conducted in the absence of any commercial or financial relationships that could be construed as a potential conflict of interest.

## Publisher’s Note

All claims expressed in this article are solely those of the authors and do not necessarily represent those of their affiliated organizations, or those of the publisher, the editors and the reviewers. Any product that may be evaluated in this article, or claim that may be made by its manufacturer, is not guaranteed or endorsed by the publisher.

## References

[1] World Health Organization . COVID-19 Weekly Epidemiological Update, edition 45. World Health Organization (2021).

[2] Tian Y Qiu X Wang C Zhao J Jiang X Niu W . Cancer Associates With Risk and Severe Events of COVID-19: A Systematic Review and Meta-Analysis. Int J Cancer (2021) 148(2):363–74. doi: 10.1002/ijc.33213 PMC7404763 32683687

[3] Lee LY Cazier J-B Angelis V Arnold R Bisht V Campton NA . COVID-19 Mortality in Patients With Cancer on Chemotherapy or Other Anticancer Treatments: A Prospective Cohort Study. Lancet (London England) (2020) 395(10241):1919–26. doi: 10.1016/S0140-6736(20)31173-9 PMC7255715 32473682

[4] Lee LYW Cazier J-B Starkey T Briggs SEW Arnold R Bisht V . COVID-19 Prevalence and Mortality in Patients With Cancer and the Effect of Primary Tumour Subtype and Patient Demographics: A Prospective Cohort Study. Lancet Oncol (2020) 21(10):1309–16. doi: 10.1016/S1470-2045(20)30442-3 PMC7444972 32853557

[5] Mato AR Roeker LE Lamanna N Allan JN Leslie L Pagel JM . Outcomes of COVID-19 in Patients With CLL: A Multicenter International Experience. Blood (2020) 136(10):1134–43. doi: 10.1182/blood.2020006965 PMC7472711 32688395

[6] Sette A Crotty S . Adaptive Immunity to SARS-CoV-2 and COVID-19. Cell (2021) 184(4):861–80. doi: 10.1016/j.cell.2021.01.007 PMC7803150 33497610

[7] Derosa L Melenotte C Griscelli F Gachot B Marabelle A Kroemer G . The Immuno-Oncological Challenge of COVID-19. Nat Cancer (2020) 1(10):946–64. doi: 10.1038/s43018-020-00122-3 35121872

[8] Mansi L Spehner L Daguindau E Bouiller K Almotlak H Stein U . Study of the SARS-CoV-2-Specific Immune T-Cell Responses in COVID-19-Positive Cancer Patients. Eur J Cancer (2021) 150:1–9. doi: 10.1016/j.ejca.2021.03.033 33882374 PMC7997727

[9] Taborska P Strizova Z Stakheev D Sojka L Bartunkova J Smrz D . CD4(+) T Cells of Prostate Cancer Patients Have Decreased Immune Responses to Antigens Derived From SARS-CoV-2 Spike Glycoprotein. Front Immunol (2021) 12:629102. doi: 10.3389/fimmu.2021.629102 34012431 PMC8128251

[10] Bange EM Han NA Wileyto P Kim JY Gouma S Robinson J . CD8+ T Cells Contribute to Survival in Patients With COVID-19 and Hematologic Cancer. Nat Med (2021) 27(7):1280–9. doi: 10.1038/s41591-021-01386-7 PMC8291091 34017137

[11] Guven DC Aktas BY Aksun MS Ucgul E Sahin TK Yildirim HC . COVID-19 Pandemic: Changes in Cancer Admissions. BMJ Support Palliat Care (2020) doi: 10.1136/bmjspcare-2020-002468 32665259

[12] Madhi SA Baillie V Cutland CL Voysey M Koen AL Fairlie L . Efficacy of the ChAdOx1 Ncov-19 Covid-19 Vaccine Against the B.1.351 Variant. N Engl J Med (2021) 384(20):1885–98. doi: 10.1056/NEJMoa2102214 PMC7993410 33725432

[13] Baden LR El Sahly HM Essink B Kotloff K Frey S Novak R . Efficacy and Safety of the mRNA-1273 SARS-CoV-2 Vaccine. N Engl J Med (2020) 384(5):403–16. doi: 10.1056/NEJMoa2035389 PMC7787219 33378609

[14] Polack FP Thomas SJ Kitchin N Absalon J Gurtman A Lockhart S . Safety and Efficacy of the BNT162b2 mRNA Covid-19 Vaccine. N Engl J Med (2020) 383(27):2603–15. doi: 10.1056/NEJMoa2034577 PMC7745181 33301246

[15] Mathieu E Ritchie H Ortiz-Ospina E Roser M Hasell J Appel C . A Global Database of COVID-19 Vaccinations. Nat Hum Behav (2021) 5(7):947–53. doi: 10.1038/s41562-021-01122-8 33972767

[16] Ritchie H Ortiz-Ospina E Beltekian D Mathieu E Hasell J Macdonald B . Coronavirus Pandemic (COVID-19). World Data (2020). 10.1038/s41597-020-00688-8 PMC7545176 33033256

[17] Garassino MC Vyas M De Vries E Kanesvaran R Giuliani R Peters S . The ESMO Call to Action on COVID-19 Vaccinations and Patients With Cancer: Vaccinate. Monitor. Educate. Ann Oncol (2021) 32(5):579–81. doi: 10.1016/j.annonc.2021.01.068 PMC7879154 33582237

[18] Ribas A Sengupta R Locke T Zaidi SK Campbell KM Carethers JM . Priority COVID-19 Vaccination for Patients With Cancer While Vaccine Supply is Limited. Cancer Discov (2021) 11(2):233–6. doi: 10.1158/2159-8290.CD-20-1817 PMC8053003 33355178

[19] Desai A Gainor JF Hegde A Schram AM Curigliano G Pal S . COVID-19 Vaccine Guidance for Patients With Cancer Participating in Oncology Clinical Trials. Nat Rev Clin Oncol (2021) 18(5):313–9. doi: 10.1038/s41571-021-00487-z PMC7957448 33723371

[20] Mandal A Singh P Samaddar A Singh D Verma M Rakesh A . Vaccination of Cancer Patients Against COVID-19: Towards the End of a Dilemma. Med Oncol (Northwood London England) (2021) 38(8):92. doi: 10.1007/s12032-021-01540-8 PMC8263320 34235592

[21] Loulergue P Alexandre J Iurisci I Grabar S Medioni J Ropert S . Low Immunogenicity of Seasonal Trivalent Influenza Vaccine Among Patients Receiving Docetaxel for a Solid Tumour: Results of a Prospective Pilot Study. Br J Cancer (2011) 104(11):1670–4. doi: 10.1038/bjc.2011.142 PMC3111157 21540859

[22] Palich R Veyri M Marot S Vozy A Gligorov J Maingon P . Weak Immunogenicity After a Single Dose of SARS-CoV-2 mRNA Vaccine in Treated Cancer Patients. Ann Oncol (2021) 32(8):1051–3. doi: 10.1016/j.annonc.2021.04.020 PMC8081573 33932501

[23] Moher D Liberati A Tetzlaff J Altman DG . Preferred Reporting Items for Systematic Reviews and Meta-Analyses: The PRISMA Statement. BMJ (2009) 339:b2535. doi: 10.1371/journal.pmed.1000097 19622551 PMC2714657

[24] Herishanu Y Avivi I Aharon A Shefer G Levi S Bronstein Y . Efficacy of the BNT162b2 mRNA COVID-19 Vaccine in Patients With Chronic Lymphocytic Leukemia. Blood (2021) 137(23):3165–73. doi: 10.1182/blood.2021011568 PMC8061088 33861303

[25] Massarweh A Eliakim-Raz N Stemmer A Levy-Barda A Yust-Katz S Zer A . Evaluation of Seropositivity Following BNT162b2 Messenger RNA Vaccination for SARS-CoV-2 in Patients Undergoing Treatment for Cancer. JAMA Oncol (2021) 7(8):1133–40. doi: 10.1001/jamaoncol.2021.2155 PMC8164144 34047765

[26] Van Oekelen O Gleason CR Agte S Srivastava K Beach KF Aleman A . Highly Variable SARS-CoV-2 Spike Antibody Responses to Two Doses of COVID-19 RNA Vaccination in Patients With Multiple Myeloma. Cancer Cell (2021) 39(8):1028–30. doi: 10.1016/j.ccell.2021.06.014 PMC8238657 34242572

[27] Barrière J Chamorey E Adjtoutah Z Castelnau O Mahamat A Marco S . Impaired Immunogenicity of BNT162b2 Anti-SARS-CoV-2 Vaccine in Patients Treated for Solid Tumors. Ann Oncol (2021) 32(8):1053–5. doi: 10.1016/j.annonc.2021.04.019 PMC8080507 33932508

[28] Monin L Laing AG Muñoz-Ruiz M McKenzie DR del Molino del Barrio I Alaguthurai T . Safety and Immunogenicity of One *Versus* Two Doses of the COVID-19 Vaccine BNT162b2 for Patients With Cancer: Interim Analysis of a Prospective Observational Study. Lancet Oncol (2021) 22(6):765–78. doi: 10.1016/S1470-2045(21)00213-8 PMC8078907 33930323

[29] Pimpinelli F Marchesi F Piaggio G Giannarelli D Papa E Falcucci P . Fifth-Week Immunogenicity and Safety of Anti-SARS-CoV-2 BNT162b2 Vaccine in Patients With Multiple Myeloma and Myeloproliferative Malignancies on Active Treatment: Preliminary Data From a Single Institution. J Hematol Oncol (2021) 14(1):81. doi: 10.1186/s13045-021-01090-6 34001183 PMC8128283

[30] Avivi I Balaban R Shragai T Sheffer G Morales M Aharon A . Humoral Response Rate and Predictors of Response to BNT162b2 mRNA COVID19 Vaccine in Patients With Multiple Myeloma. Br J Haematol (2021) 195(2):186–93. doi: 10.1111/bjh.17608 PMC8444771 34196388

[31] Herzog Tzarfati K Gutwein O Apel A Rahimi-Levene N Sadovnik M Harel L . BNT162b2 COVID-19 Vaccine is Significantly Less Effective in Patients With Hematologic Malignancies. Am J Hematol (2021) 96(10):1195–203. doi: 10.1002/ajh.26284 PMC8420332 34185336

[32] Shroff RT Chalasani P Wei R Pennington D Quirk G Schoenle MV . Immune Responses to COVID-19 mRNA Vaccines in Patients With Solid Tumors on Active, Immunosuppressive Cancer Therapy. MedRxiv: Preprint Server Health Sci (2021). doi: 10.1101/2021.05.13.21257129

[33] Addeo A Shah PK Bordry N Hudson RD Albracht B Di Marco M . Immunogenicity of SARS-CoV-2 Messenger RNA Vaccines in Patients With Cancer. Cancer Cell (2021) 39(8):1091–98.e2. doi: 10.1016/j.ccell.2021.06.009 PMC8218532 34214473

[34] Goshen-Lago T Waldhorn I Holland R Szwarcwort-Cohen M Reiner-Benaim A Shachor-Meyouhas Y . Serologic Status and Toxic Effects of the SARS-CoV-2 BNT162b2 Vaccine in Patients Undergoing Treatment for Cancer. JAMA Oncol (2021) 7(10):1507–13. doi: 10.1001/jamaoncol.2021.2675 PMC8267843 34236381

[35] Gavriatopoulou M Terpos E Kastritis E Briasoulis A Gumeni S Ntanasis-Stathopoulos I . Low Neutralizing Antibody Responses in WM, CLL and NHL Patients After the First Dose of the BNT162b2 and AZD1222 Vaccine. Clin Exp Med (2021), 1–5. doi: 10.1007/s10238-021-00746-4 34283338 PMC8290394

[36] Terpos E Zagouri F Liontos M Sklirou AD Koutsoukos K Markellos C . Low Titers of SARS-CoV-2 Neutralizing Antibodies After First Vaccination Dose in Cancer Patients Receiving Checkpoint Inhibitors. J Hematol Oncol (2021) 14(1):86. doi: 10.1186/s13045-021-01099-x 34059088 PMC8165511

[37] Chowdhury O Bruguier H Mallett G Sousos N Crozier K Allman C . Impaired Antibody Response to COVID-19 Vaccination in Patients With Chronic Myeloid Neoplasms. Br J Haematol (2021) 194(6):1010–5. doi: 10.1111/bjh.17644 PMC8444839 34132395

[38] Terpos E Trougakos IP Gavriatopoulou M Papassotiriou I Sklirou AD Ntanasis-Stathopoulos I . Low Neutralizing Antibody Responses Against SARS-CoV-2 in Older Patients With Myeloma After the First BNT162b2 Vaccine Dose. Blood (2021) 137(26):3674–6. doi: 10.1182/blood.2021011904 PMC8061093 33861315

[39] Palich R Veyri M Vozy A Marot S Gligorov J Benderra MA . High Seroconversion Rate But Low Antibody Titers After Two Injections of BNT162b2 (Pfizer-BioNTech) Vaccine in Patients Treated With Chemotherapy for Solid Cancers. Ann Oncol: Off J Eur Soc Med Oncol (2021) 32(10):1294–5. doi: 10.1016/j.annonc.2021.06.018 PMC8217700 34171494

[40] Thakkar A Gonzalez-Lugo JD Goradia N Gali R Shapiro LC Pradhan K . Seroconversion Rates Following COVID-19 Vaccination Among Patients With Cancer. Cancer Cell (2021) 39(8):1081–90.e2. doi: 10.1016/j.ccell.2021.06.002 PMC8179248 34133951

[41] Fong D Mair MJ Mitterer M . High Levels of Anti-SARS-CoV-2 IgG Antibodies in Previously Infected Patients With Cancer After a Single Dose of BNT 162b2 Vaccine. Eur J Cancer (2021) 154:4–6. doi: 10.1016/j.ejca.2021.05.036 34217909 PMC8192959

[42] Bird S Panopoulou A Shea RL Tsui M Saso R Sud A . Response to First Vaccination Against SARS-CoV-2 in Patients With Multiple Myeloma. Lancet Haematol (2021) 8(6):e389–92. doi: 10.1016/S2352-3026(21)00110-1 PMC8055205 33887255

[43] Re D Barrière J Chamorey E Delforge M Gastaud L Petit E . Low Rate of Seroconversion After mRNA Anti-SARS-CoV-2 Vaccination in Patients With Hematological Malignancies. Leuk Lymphoma (2021) 1–3. doi: 10.1080/10428194.2021.1957877 34308748

[44] Lim SH Campbell N Johnson M Joseph-Pietras D Collins GP O’Callaghan A . Antibody Responses After SARS-CoV-2 Vaccination in Patients With Lymphoma. Lancet Haematol (2021) 8(8):e542–4. doi: 10.1016/S2352-3026(21)00199-X PMC8253538 34224667

[45] Roeker LE Knorr DA Thompson MC Nivar M Lebowitz S Peters N . COVID-19 Vaccine Efficacy in Patients With Chronic Lymphocytic Leukemia. Leukemia (2021) 35(9):2703–5. doi: 10.1038/s41375-021-01270-w PMC8118367 33986431

[46] Agha M Blake M Chilleo C Wells A Haidar G . Suboptimal Response to COVID-19 mRNA Vaccines in Hematologic Malignancies Patients. medRxiv (2021). doi: 10.1101/2021.04.06.21254949 PMC8320282 34337100

[47] Diefenbach C Caro J Koide A Grossbard M Goldberg JD Raphael B . Impaired Humoral Immunity to SARS-CoV-2 Vaccination in Non-Hodgkin Lymphoma and CLL Patients. MedRxiv: Preprint Server Health Sci (2021). doi: 10.1101/2021.06.02.21257804

[48] Harrington P de Lavallade H Doores KJ O’Reilly A Seow J Graham C . Single Dose of BNT162b2 mRNA Vaccine Against SARS-CoV-2 Induces High Frequency of Neutralising Antibody and Polyfunctional T-Cell Responses in Patients With Myeloproliferative Neoplasms. Leukemia (2021). doi: 10.1038/s41375-021-01300-7 PMC8140572 34023850

[49] Harrington P Doores KJ Radia D O’Reilly A Lam HPJ Seow J . Single Dose of BNT162b2 mRNA Vaccine Against Severe Acute Respiratory Syndrome Coronavirus-2 (SARS-CoV-2) Induces Neutralising Antibody and Polyfunctional T-Cell Responses in Patients With Chronic Myeloid Leukaemia. Br J Haematol (2021) 194(6):999–1006. doi: 10.1111/bjh.17568 34085278 PMC8239833

[50] Zhang Z Liu S Zhang B Qiao L Zhang Y Zhang Y . T Cell Dysfunction and Exhaustion in Cancer. Front Cell Dev Biol (2020) 8(17). doi: 10.3389/fcell.2020.00017 PMC7027373 32117960

[51] Solodky ML Galvez C Russias B Detourbet P N’Guyen-Bonin V Herr AL . Lower Detection Rates of SARS-COV2 Antibodies in Cancer Patients *Versus* Health Care Workers After Symptomatic COVID-19. Ann Oncol (2020) 31(8):1087–8. doi: 10.1016/j.annonc.2020.04.475 PMC7252166 32360743

[52] Marra A Generali D Zagami P Cervoni V Gandini S Venturini S . Seroconversion in Patients With Cancer and Oncology Health Care Workers Infected by SARS-CoV-2. Ann Oncol (2021) 32(1):113–9. doi: 10.1016/j.annonc.2020.10.473 PMC7577226 33098994

[53] Thakkar A Pradhan K Jindal S Cui Z Rockwell B Shah AP . Patterns of Seroconversion for SARS-CoV-2 IgG in Patients With Malignant Disease and Association With Anticancer Therapy. Nat Cancer (2021) 2(4):392–9. doi: 10.1038/s43018-021-00191-y PMC8519533 34661163

[54] Pilishvili T Fleming-Dutra KE Farrar JL Gierke R Mohr NM Talan DA . Interim Estimates of Vaccine Effectiveness of Pfizer-BioNTech and Moderna COVID-19 Vaccines Among Health Care Personnel—33 US Sites, January–March 2021. Morb Mortal Wkly Rep (2021) 70(20):753. doi: 10.15585/mmwr.mm7020e2 PMC8136422 34014909

[55] Sadoff J Gray G Vandebosch A Cárdenas V Shukarev G Grinsztejn B . Safety and Efficacy of Single-Dose Ad26.Cov2.S Vaccine Against Covid-19. N Engl J Med (2021) 384(23):2187–201. doi: 10.1056/NEJMoa2101544 PMC8220996 33882225

[56] O’Brien MP Forleo-Neto E Musser BJ Isa F Chan K-C Sarkar N . Subcutaneous REGEN-COV Antibody Combination to Prevent Covid-19. N Engl J Med (2021) 385(13):1184–95. doi: 10.1056/NEJMoa2109682 PMC8362593 34347950

[57] Re D Seitz-Polski B Carles M Brglez V Graça D Benzaken S . Humoral and Cellular Responses After a Third Dose of BNT162b2 Vaccine in Patients Treated for Lymphoid Malignancies. medRxiv (2021). doi: 10.1101/2021.07.18.21260669 PMC8844396 35165284

[58] Werbel WA Boyarsky BJ Ou MT Massie AB Tobian AAR Garonzik-Wang JM . Safety and Immunogenicity of a Third Dose of SARS-CoV-2 Vaccine in Solid Organ Transplant Recipients: A Case Series. Ann Intern Med (2021) 174(9):1330–2. doi: 10.1001/jama.2021.4385 PMC8252023 34125572

[59] Hill JA Ujjani CS Greninger AL Shadman M Gopal AK . Immunogenicity of a Heterologous COVID-19 Vaccine After Failed Vaccination in a Lymphoma Patient. Cancer Cell (2021) 39(8):1037–8. doi: 10.1016/j.ccell.2021.06.015 PMC8233960 34242571

[60] Pan H Wu Q Zeng G Yang J Jiang D Deng X . Immunogenicity and Safety of a Third Dose, and Immune Persistence of CoronaVac Vaccine in Healthy Adults Aged 18-59 Years: Interim Results From a Double-Blind, Randomized, Placebo-Controlled Phase 2 Clinical Trial. medRxiv (2021). doi: 10.1101/2021.07.23.21261026

[61] Rubin LG Levin MJ Ljungman P Davies EG Avery R Tomblyn M . 2013 IDSA Clinical Practice Guideline for Vaccination of the Immunocompromised Host. Clin Infect Dis (2014) 58(3):e44–100. doi: 10.1093/cid/cit684 24311479

[62] Rieger CT Liss B Mellinghoff S Buchheidt D Cornely OA Egerer G . Anti-Infective Vaccination Strategies in Patients With Hematologic Malignancies or Solid Tumors-Guideline of the Infectious Diseases Working Party (AGIHO) of the German Society for Hematology and Medical Oncology (DGHO). Ann Oncol: Off J Eur Soc Med Oncol (2018) 29(6):1354–65. doi: 10.1093/annonc/mdy117 PMC6005139 29688266

[63] Kamar N Abravanel F Marion O Couat C Izopet J Del Bello A . Three Doses of an mRNA Covid-19 Vaccine in Solid-Organ Transplant Recipients. N Engl J Med (2021) 385(7):661–2. doi: 10.1056/NEJMc2108861 PMC8262620 34161700

[64] Hall VG Ferreira VH Ku T Ierullo M Majchrzak-Kita B Chaparro C . Randomized Trial of a Third Dose of mRNA-1273 Vaccine in Transplant Recipients. N Engl J Med (2021) 385(13):1244–6. doi: 10.1056/NEJMc2111462 PMC8385563 34379917

[65] In Brief: Third Dose of mRNA-Based COVID-19 Vaccines for Immunocompromised Persons. Med Lett Drugs Ther (2021) 63(1633):145–6. 34550960

[66] Jin P Li J Pan H Wu Y Zhu F . Immunological Surrogate Endpoints of COVID-2019 Vaccines: The Evidence We Have *Versus* the Evidence We Need. Signal Transduct Target Ther (2021) 6(1):48. doi: 10.1038/s41392-021-00481-y 33531462 PMC7851657

[67] Le Bert N Tan AT Kunasegaran K Tham CYL Hafezi M Chia A . SARS-CoV-2-Specific T Cell Immunity in Cases of COVID-19 and SARS, and Uninfected Controls. Nature (2020) 584(7821):457–62. doi: 10.1038/s41586-020-2550-z 32668444

[68] Deng Y Li Y Yang R Tan W . SARS-CoV-2-Specific T Cell Immunity to Structural Proteins in Inactivated COVID-19 Vaccine Recipients. Cell Mol Immunol (2021) 18(8):2040–1. doi: 10.1038/s41423-021-00730-8 PMC8280564 34267334

[69] Sahin U Muik A Vogler I Derhovanessian E Kranz LM Vormehr M . BNT162b2 Vaccine Induces Neutralizing Antibodies and Poly-Specific T Cells in Humans. Nature (2021) 595(7868):572–7. doi: 10.1038/s41586-021-03653-6 34044428

[70] Guevara-Hoyer K Fuentes-Antrás J de la Fuente-Muñoz E Rodríguez de la Peña A Viñuela M Cabello-Clotet N . Serological Tests in the Detection of SARS-CoV-2 Antibodies. Diagnostics (2021) 11(4):678. doi: 10.3390/diagnostics11040678 33918840 PMC8069538

[71] Falzone L Gattuso G Tsatsakis A Spandidos DA Libra M Falzone L . Current and Innovative Methods for the Diagnosis of COVID−19 Infection (Review). Int J Mol Med (2021) 47(6):100. doi: 10.3892/ijmm.2021.4933 33846767 PMC8043662

[72] Theel ES Slev P Wheeler S Couturier MR Wong SJ Kadkhoda K . The Role of Antibody Testing for SARS-CoV-2: Is There One? J Clin Microbiol (2020) 58(8):e00797–20. doi: 10.1128/JCM.00797-20 PMC7383527 32350047

[73] James J Rhodes S Ross CS Skinner P Smith SP Shipley R . Comparison of Serological Assays for the Detection of SARS-CoV-2 Antibodies. Viruses (2021) 13(4):713. doi: 10.3390/v13040713 33924168 PMC8074400

[74] Coste AT Jaton K Papadimitriou-Olivgeris M Greub G Croxatto A . Comparison of SARS-CoV-2 Serological Tests With Different Antigen Targets. J Clin Virol (2021) 134:104690. doi: 10.1016/j.jcv.2020.104690 33253926 PMC7670982

[75] Lopez Bernal J Andrews N Gower C Gallagher E Simmons R Thelwall S . Effectiveness of Covid-19 Vaccines Against the B.1.617.2 (Delta) Variant. N Engl J Med (2021) 385(7):585–94. doi: 10.1056/NEJMoa2108891 PMC8314739 34289274

[76] Vivarelli S Falzone L Grillo CM Scandurra G Torino F Libra M . Cancer Management During COVID-19 Pandemic: Is Immune Checkpoint Inhibitors-Based Immunotherapy Harmful or Beneficial? Cancers (2020) 12(8):2237. doi: 10.3390/cancers12082237 PMC7465907 32785162

[77] Yekedüz E Dursun B Aydın GÇ Yazgan SC Öztürk HH Azap A . Clinical Course of COVID-19 Infection in Elderly Patient With Melanoma on Nivolumab. J Oncol Pharm Pract (2020) 26(5):1289–94. doi: 10.1177/1078155220924084 32423324

[78] Vivarelli S Falzone L Torino F Scandurra G Russo G Bordonaro R . Immune-Checkpoint Inhibitors From Cancer to COVID−19: A Promising Avenue for the Treatment of Patients With COVID−19 (Review). Int J Oncol (2021) 58(2):145–57. doi: 10.3892/ijo.2020.5159 PMC7864014 33491759

[79] Terpos E Gavriatopoulou M Ntanasis-Stathopoulos I Briasoulis A Gumeni S Malandrakis P . The Neutralizing Antibody Response Post COVID-19 Vaccination in Patients With Myeloma is Highly Dependent on the Type of Anti-Myeloma Treatment. Blood Cancer J (2021) 11(8):138. doi: 10.1038/s41408-021-00530-3 34341335 PMC8327056

[80] Singer J Le N-S Mattes D Klamminger V Hackner K Kolinsky N . Evaluation of Antibody Responses to COVID-19 Vaccines Among Solid Tumor and Hematologic Patients. Cancers (Basel) (2021) 13(17):4312. doi: 10.3390/cancers13174312 34503127 PMC8430869

[81] Reynolds CJ Pade C Gibbons JM Butler DK Otter AD Menacho K . Prior SARS-CoV-2 Infection Rescues B and T Cell Responses to Variants After First Vaccine Dose. Science (2021) 372(6549):1418. doi: 10.1126/science.abh1282 PMC8168614 33931567

[82] Teijaro JR Farber DL . COVID-19 Vaccines: Modes of Immune Activation and Future Challenges. Nat Rev Immunol (2021) 21(4):195–7. doi: 10.1038/s41577-021-00526-x PMC7934118 33674759

