# Supplementary figures and images for: Antibody Responses to COVID-19 Vaccination in Cancer: A Systematic Review
Source: Front Oncol. 2021 Nov 4;11:759108. doi: 10.3389/fonc.2021.759108 (PMC8599356; doi:10.3389/fonc.2021.759108)

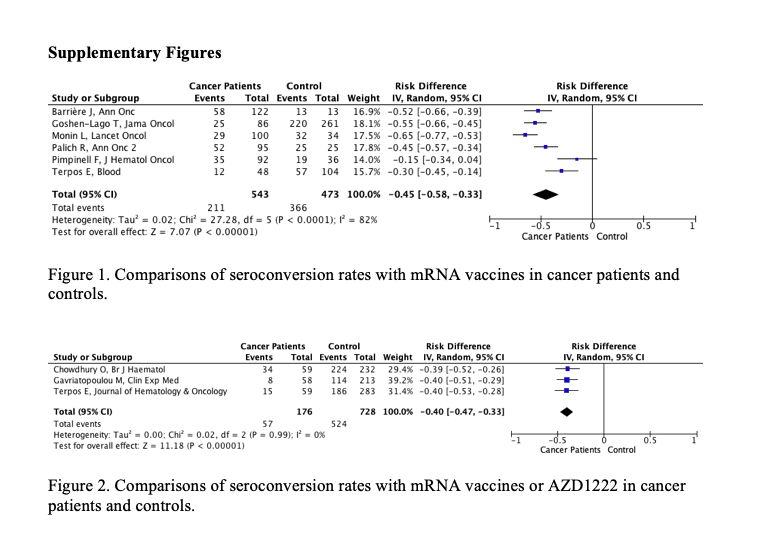

Supplement: Supplementary file 1 [file Image_1.png]
